# Uterine Fibroids and Diet

**DOI:** 10.3390/ijerph18031066

**Published:** 2021-01-25

**Authors:** Andrea Tinelli, Marina Vinciguerra, Antonio Malvasi, Mladen Andjić, Ivana Babović, Radmila Sparić

**Affiliations:** 1 Department of Obstetrics and Gynecology, “Veris delli Ponti” Hospital, Scorrano, 73020 Lecce, Italy; 2 Division of Experimental Endoscopic Surgery, Imaging, Technology and Minimally Invasive Therapy, Vito Fazzi Hospital, 73100 Lecce, Italy; 3 Laboratory of Human Physiology, Phystech BioMed School, Faculty of Biological & Medical Physics, Moscow Institute of Physics and Technology (State University), 141701 Moscow, Russia; antoniomalvasi@gmail.com; 4 Department of Biomedical and Human Oncological Science (DIMO), 1st Unit of Obstetrics and Gynecology, School of Medicine, University of Bari Aldo Moro, 70124 Bari, Italy; marinavinciguerra@icloud.com; 5 Department of Obstetrics & Gynecology, Santa Maria Hospital GVM Care & Research, 70124 Bari, Italy; 6 Clinic for Gynecology and Obstetrics, Clinical Centre of Serbia, Višegradska 26, 11000 Belgrade, Serbia; andjicmladen94@gmail.com (M.A.); ivana.r.babovic@gmail.com (I.B.); radmila@rcub.bg.ac.rs (R.S.); 7 School of Medicine, University of Belgrade, 11000 Belgrade, Serbia

**Keywords:** uterine fibroids, myoma, diet, nutrition, dietary habits, fruit, vegetable, vitamin D

## Abstract

Uterine myomas or fibroids are the most common benign female tumors of the reproductive organs, associated with significant morbidity and quality of life impairment. Several epidemiological risk factors for their occurrence have been identified so far, including nutrition and dietary habits. In this investigation, authors reviewed, as a narrative review, the data about diet and uterine myoma development in order to homogenize the current data. A PubMed search was conducted for the years 1990–2020, using a combination of keywords of interest for the selected topic. The authors searched the databases, selecting the randomized clinical studies, the observational studies, and the basic (experimental), clinical, and epidemiological researches. Once they collected the articles, they analyzed them according to the number of citations of each article, starting from the most cited to the least cited articles. Subsequently, authors collected the data of each article and inserted them in the various research paragraphs, summarizing the data collected. In this way, they crossed the available data regarding the association between nutrition habits and dietary components and myoma onset and growth. Many nutrients and dietary habits are associated with myoma development risk. These factors include low intakes of fruit, vegetables, and vitamin D, as well as pollutants in food. Despite the available data on the influence of some foods on the development of fibroids, further research is mandatory to understand all the nutrition risk factors which contribute to myoma growth and how exactly these risk factors influence myoma pathogenesis.

## 1. Introduction

Uterine myomas represent the most frequent form of benign tumors of the female reproductive organs [1], representing one of the major women’s health issues worldwide which have health and socio-economic consequences. They are monoclonal tumors of uterine smooth muscle; thus, they originate from the myometrial stem cells and contain a large amount of extracellular matrix containing collagen, fibronectin, and proteoglycans [2,3]. Uterine myomas represent a significant source of morbidity for women of reproductive age. They occasionally cause heavy or prolonged menstrual bleeding which can lead to iron-deficiency anemia and social embarrassment. The presence of an enlarged uterus caused by uterine myomas may also cause abdominal distension, pain, gastrointestinal symptoms (such as diarrhea or constipation), and urinary symptoms (such as urinary frequency, urinary retention, or nocturia). Nevertheless, many women remain asymptomatic regardless of myoma size [4].

Even though uterine myomas represent a significant global health problem, the morbidity they cause is underscored by hysterectomy which represents uterine myoma’s main treatment option, a major surgical procedure which eliminates childbearing possibilities and has numerous sequelae for general health. Besides the hysterectomy, there are other options for uterine fibroid management. Fibroid management is also based on additional options, which include myomectomy, thermo-ablative therapies, blood vessel embolization procedures, magnetic resonance-guided focused ultrasounds (MRgFUSs), and symptomatic medical therapies (i.e., progesterone receptor modulators, tranexamic acid, gonadotropin-releasing hormone agonists, and mainly both the oral contraceptive and the levonorgestrel intrauterine device) [3]. The need to preserve female fertility and the myoma’s own characteristics mainly guide the therapeutic choice of the treatments for uterine myomas [5].

Health care expenses and myoma-related indirect costs, such as the cost of sanitary products, complementary and alternative therapies, and the loss of monetary income due to disability and time out of work represent major social and economic problems [6]. Moreover, infertility and pregnancy complications linked to fibroids could be added to the overall costs and morbidity [6]. Many epidemiological factors are linked to the onset and the development of uterine myomas, but the exact cause and mechanisms of uterine myoma’s development and growth are not fully understood. The possible mechanisms of uterine myomas pathogenesis are presented in Diagram 1, even if there are conflicting data about the pathogenesis. It has been assumed that uterine myomas development could be linked with predisposing risk factors, genetic mechanisms, and effectors and promoters [7]. There are several theories which explain the initiators of fibroids. It has been stated that increased levels of estrogen and progesterone lead to an increase of the mitotic rate which is responsible for somatic mutation [8]. Increased concentrations of estrogen receptors have been found in non-neoplastic myometrium regions of myomatous uteruses [9]. There is theory that uterine myoma pathogenesis is similar to a response to injury with the synthesis of the extracellular fibrous matrix [10]; ischemic damage could be linked to the increased synthesis of vasoconstrictive substances at the time of the menses [11]. On the other hand, vascular damage led to an overexpression of basic fibroblast growth factors in leiomyomas [12,13]. Epidemiology shows a connection between myoma growth and risk factors, such as age, race, heritage, reproductive factors, sex hormones, hypertension, and infection [14]. According to epidemiological evidence, specific dietary components and nutrition have the potential ability to influence hormone-related diseases and thus possibly also fibroid formation and growth [15,16].

The role of estrogen in the disease is relevant in this context. Accordingly, food components and dietary habits may be linked with the risk of developing uterine myoma. Indeed, pollutants detected in food such as fruit, vegetables, and fish may promote some hormone-related diseases [17,18]. The data about the relationship between uterine myoma risk and food components and dietary habits are conflicting. Thus, further investigation about nutrition factors which may contribute to uterine myoma development and the exact mechanisms of their influence on myoma onset and growth are necessary.

**Figure 1:**
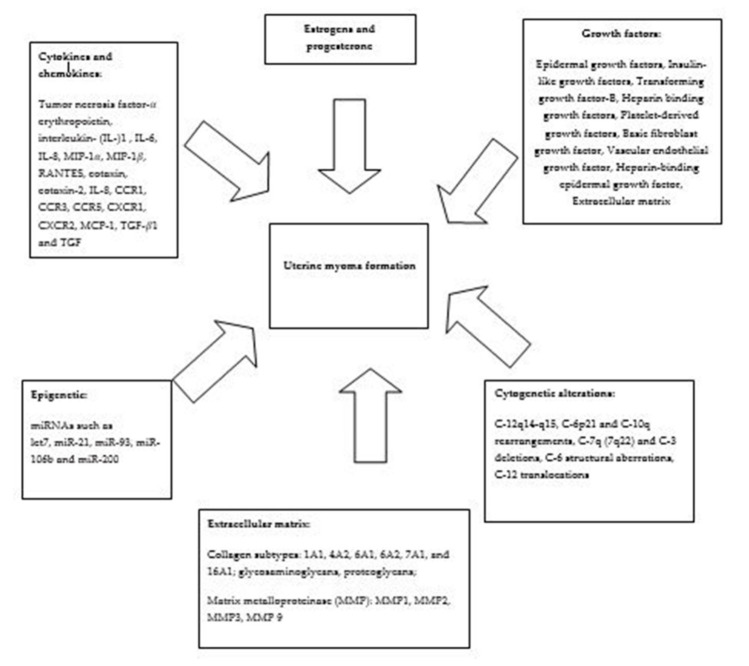
Possible Mechanism of uterine myoma’s pathogenesis.

This article reviews the studies concerning diet and uterine myoma risk, discussing some of the most important findings of the potential relationship between uterine myoma risk and diet, identifying potentially modifiable risk factors of myoma development. (Figure 1).

## 2. Materials and Methods

In this review article, authors investigated the available data regarding the association between nutrition habits and dietary components and myoma onset and growth. We performed a selected PubMed search to reduce bias with other databases, for the years 1990–2020, using a combination of keywords such as “myoma”, “leiomyoma”, “fibromyoma”, “leiomyofibroma”, “fibroleiomyoma”, “fibroid”, “myomectomy”, “diet”, “nutrition”, “fat”, “supplements”, “tea”, “vegetable”, “meat”, “fish”, “dairy”, “fruit” “alcohol”, and “vitamins”. The authors searched the database, selecting the randomized clinical studies, the observational studies, and the basic (experimental), clinical, and epidemiological researches. Once we collected the articles, we analyzed them according to the number of citations of each article, starting from the most cited to the least cited articles. The terms “leiomyomas”, “fibroids”, “fibromyomas”, “leiomyofibromas”, and “fibroleiomyomas” can also be found in the literature describing myomas [19], but in the review, the term myoma was preferred. Subsequently, we collected the data of each article and inserted them in the research paragraphs, summarizing the data collected. This narrative review aims to provide information regarding the influence of diet on uterine myoma risk (Table 1) and pathogenesis, with references to role of natural compounds (Table 2) and pollutants in food.

## 3. Results

### 3.1. Glycemic Index, Dietary Fibers and Cereals Intake and Uterine Myoma

According to the results of the Black Women’s Health Study (BWHS), high dietary glycemic index (GI) and glycemic load (GL) may be associated with an increased uterine myoma risk in some women for hormone-responsive tumors, such as endometrial and ovarian cancers, through a common mechanism [20]. Both GI and GL may stimulate tumorigenesis by increasing the bioavailability of estradiol or endogenous concentrations of insulin-like growth factor I (IGF-I), which in vitro promotes the proliferation of myoma cells, increasing the display of IGF-I gene expression and protein synthesis. Epidemiologic studies showed that a high GL is a risk factor for endometrial and ovarian cancers, which, like uterine myoma, are hormone-responsive tumors [20]. Instead, the BWHS and a Japanese study did not find a significant relationship between a high-fiber diet, rich in whole foods, and myoma development, as was also confirmed by Chinese and Italian case-control studies [20,21,22,23]. Similar results were reported from Chinese and Italian case-control studies investigating the association between whole-grain food intake and myoma. These studies failed to prove a significant relationship between cereal intake and myoma prevalence [21,22,23].

### 3.2. Soya Intake and Uterine Myoma

The possible influence of soy intake on uterine myoma pathogenesis is still controversial. The literature is not unique on the conclusions, as some studies disagree [20,21,24,25], but many others have found a considerable statistical significance [26,27]. Some interesting investigations have focused on early feeding with soy products, as in two studies conducted in Chinese Han women. They demonstrated a statistically significant relationship between uterine myoma and soy and soy milk consumption [28,29,30,31]. Interestingly, these results are from studies that have researched the influence of early feeding with soy products on uterine myoma pathogenesis [28,29,30,31]. Even if the BWHS showed a lack of influence on myoma onset [29], conversely, other studies conducted in white non-Hispanic and African-American women have reported a significantly higher uterine myoma risk in relation to early-life soy products exposure [30,31]. This link is refuted by the Study of Environment, Lifestyle, and Fibroids (SELF), according to which, however, it was that revealed soy intake positively correlated with the myoma volume. In fact, soy phytoestrogens, especially isoflavones, are compounds with both a mammalian estrogen-like structure and a weak estrogenic effect, thus they may act as antiestrogens by competing with estrogen receptor binding or altering estrogen biosynthesis [32].

### 3.3. Dietary Fat, Meat and Fish Intake

Uterine myoma risk does not seem to be influenced by dietary fat, as claimed by a Japanese study [21] and confirmed by Italian and Chinese studies, which analyzed the consumption of eggs, butter, margarine, and oil [20,23]. The BWHS showed that total fat and fat subtypes are not appreciably associated with uterine myoma risk overall, although an inverse statistically significant correlation was observed for specific saturated fatty acids and a positive one for monounsaturated and polyunsaturated fatty acids. In particular, a small increase in myoma risk may be related to long-chain omega-3 fatty acid intake [25]. A five year prospective cohort study of premenopausal African American women, based on serial ultrasound exams, did not report a correlation between myoma risk and dietary total fat or each type of fatty acid and trans-fat, but surprisingly each of the three marine ω-3 polyunsaturated fatty acids (PUFAs) positively correlated uterine myoma incidence, which in fact was 49% higher in relation to docosahexaenoic acid intake [33]. Thus, although the literature attributes to ω-3 PUFAs an inhibitory effect on uterine myoma growth, these opposite epidemiological findings may be explained by persistent pollutants found in fish, mostly polychlorinated biphenyls and mercury [26]. Most recently a possible n-3 PUFAs involvement in myoma pathogenesis was suggested by a multivariable models-based study, conducted on premenopausal women and named Nurses’Health Study II, which, despite not finding any link between dietary fats and myoma, showed an inverse association between total n-3 PUFAs as well as a positive association of trans fatty acids and the onset of uterine myoma, when erythrocyte fatty acids were examined [34]. At the base there should be the estrogenic or inflammatory effects of dietary fat, as demonstrated by both a reduction of serum estradiol levels in relation to lower fat consumption among both premenopausal and postmenopausal women [35] and by a fat-related increase of T-helper cytokines, meant as a promoter either of systemic chronic inflammation or of fibrous tissue and smooth muscle growth [36]. As an example, dietary trans-fats lead to the production of proinflammatory cytokines and other inflammatory markers, which in turn lead to the secretion of enzymes in the endometrial extracellular matrix [37]. Conversely, it has been observed that polyunsaturated fatty acids are inversely associated with inflammatory cytokines [38].

It has also been observed that omega-3 fatty acids lead to membrane architecture remodeling and downregulation of the expression of genes involved in mechanical signaling and lipid accumulation in leiomyoma cells. These finding implicate that these compounds could be a preventive and/or therapeutic option for uterine myoma in the future [39].

Data about the effect of meat consumption on uterine myoma risk are contradictory. The association between meat consumption and uterine myoma risk are insignificant in the Chinese population, but significant for Italian women, mostly regarding beef, other red meat, and ham [27,30]. These contradictory results could be explained by the different dietary components in many countries. Equally contradictory are the data regarding fish intake and myoma risk, an association which is not recognized by the Chinese study [20], but reported as being an inverse association by the Italian case-control study [23]. According to the results of a cohort study conducted in the Great Lakes area in the United States of America, fish consumption was positively associated with fibroid risk, with an incidence rate ratio of 1.2 for each ten-year increment of fish consumption [40]. This heterogeneity is justified by the difference in dietary compounds and pollutants in various countries, such as Polychlorinated Biphenyls (PCBs), which are linked to myoma prevalence in animals, and whose levels vary widely in fish [41].

### 3.4. Fruit and Vegetable Intake and Uterine Myoma Risk

The literature has demonstrated an association between fruit and vegetable intake and uterine myoma risk. The results of these studies could explain the possible role of vegetable and fruit nutrition contents in the pathogenesis of uterine myomas. The BWHS evidenced that women who have four fruit or vegetable servings per day have a lower risk for developing uterine myomas, in comparison with women who have just one fruit or vegetable serving per day. The risk association was stronger for fruit (two servings/day compared with two servings/week), mostly citrus, than for vegetables (two servings/day compared with four servings/week) [22]. The same inverse association emerged from an Italian study that analyzed the relationship between dietary indicators and the risk of uterine myomas. According to the results of this study, women with uterine myomas reported a less frequent consumption of green vegetables and fruit. Similar to the results of the studies conducted in the Italian population and African-American women, two studies [20,42] performed on a Chinese population confirmed that vegetable and fruit intakes significantly decrease uterine myoma risk in premenopausal women. He et al. performed a case-control study [20] showing that fruit and vegetable intake demonstrated a protective role in uterine myoma pathogenesis in Chinese premenopausal women. Shen et al. [42] investigated, with a case-control study, the dietary habits of 1200 Chinese Han women. They evaluated 600 women with uterine myoma, enrolled by a questionnaire, who demonstrated a lower consumption of broccoli, cabbage, Chinese cabbage, tomatoes, and apples, in comparison to the 600 healthy women. Moreover, the possible role of vegetables and fruit in myoma formation could be accomplished through dietary phytochemicals. Dietary phytochemicals could modulate myoma pathogenesis, extracellular matrix deposition, cell proliferation, and angiogenesis, but further studies are needed to ascertain their therapeutic effects [43]. Extracts of some kinds of vegetables and fruit contain phytochemicals that have in vitro efficacy against uterine myoma proliferation.

Islam et al. reported that strawberry extract increased the percentage of apoptotic and dead cells, as well as increasing reactive oxygen species in leiomyoma cells. According to Islam et al. [44], strawberry extract treatment leads to the decrease of the extracellular acidification rate as well as collagen 1A1, fibronectin, and versican mRNA expression in leiomyoma cells. Similar results were reported by Giampieri et al. [45]. They reported that leiomyoma cells which are treated with the methanolic extract of Alba and Romina strawberry cultivars induce apoptosis and increase intracellular reactive oxygen species levels [46]. It has also been observed that both extracts significantly decreased collagen 1A1, fibronectin, versican, and activin A messenger RNA expression in leiomyoma cells [45]. Results obtained from both studies suggest that strawberries can be developed as a preventive and/or therapeutic agent for uterine myoma. On the other hand, it has been reported that curcumin, a nutritional supplement with antineoplastic activity, lead to leiomyoma cell apoptosis and decreased fibronectin expression [46]. It has been observed that curcumin regulate leiomyocite apoptosis, via the stimulating of caspase-3 and caspase-9 expression and the inhibition of extracellular signal-regulated kinase 1 (ERK1) and ERK2 and nuclear factor kappa-light-chain-enhancer of activated B cells (NF-κB [46]. Results observed from a study on the influence of curcumin on Eker rat-derived uterine leiomyoma cell lines suggested that the curcumin inhibitory effect on leiomyoma cell proliferation occurs through the activation of peroxisome proliferator-activated receptor-gamma (PPARγ) [47]. Dietary phytochemicals, such as quercetin and indole-3-carbinol, are recognized for their potential antiproliferative effect on leiomyoma cells. Both quercetin and indole-3-carbinol reduce the expression of fibronectin and collagen 1A1 in leiomyoma cells. It has been reported that both quercetin and indole-3-carbinol reduce the migration of leiomyoma cells, as well as myometrial cell proliferation [48]. It has also been reported that lycopene, a major carotenoid in tomatoes, leads to a decrease in the incidence and size of leiomyomas in the animal model of oviducts of Japanese quail [49]. Similar results were observed by Sahin et al. [50], showing that dietary supplementation with lycopene reduces the incidence and size of spontaneously occurring leiomyoma of the oviduct in the Japanese quail. Further clinical trials are needed to investigate the efficiency of lycopene and tomato powder supplementation in the prevention and treatment of uterine myoma in the human population. Recent studies in animal models observed that resveratrol has an inhibitory effect on the proliferation of primary human myoma cell cultures. It has been reported that resveratrol decreased myoma cell proliferation via a mechanism dependent on cross-talk between integrin αvβ3 and IGF-1R. Nevertheless, resveratrol led to an increased expression of proapoptotic genes such as cyclooxygenase (COX)-2, p21 and CDKN2 [51].

Chen et al. [52] have reported that resveratrol suppresses the growth of leiomyomas in vitro in an animal model of Eker rats, and decreased the proportion of cells showing an expression of proliferation cell nuclear antigen and α-smooth muscle actin. The same study observed that resveratrol decreases the protein expression of proliferation cell nuclear antigen, fibronectin, and upregulated the ratio of Bcl-2-associated X and B-cell lymphoma/leukemia 2 in vivo, as well as leiomyoma cell viability and mRNA levels of fibronectin and the protein expression of collagen type 1 and α-SMA (extracellular matrix protein marker), and protein levels of β-catenin [52]. These findings indicated that resveratrol reduced extracellular matrix-related protein expression in primary human leiomyoma cells, and it is a candidate it for potential anti-fibrotic therapy for uterine myoma.

### 3.5. Alcohol, Coffee, and Tea Consumption and Uterine Myoma

It is well known that alcohol, coffee, and tea consumption are associated with a higher risk of various diseases, but with regards to uterine myoma risk the available findings are still controversial. The BWHS reported a significant increase in myoma risk with 20 years or longer alcohol consumption as well as current alcohol intake, particularly beer. This study has also reported the stronger association of beer consumption with uterine myoma risk, in comparison to wine and liquor consumption [53]. The California Teachers Study (CTS) significantly correlated a daily intake of at least 20 g of alcohol with the surgical treatment of myomas [54]. What has been highlighted is precisely the opposite of the mentioned studies, as the studies failed to document a significant association between alcohol consumption and uterine myomas [20,23]. The true mechanisms which link alcohol intake and myoma risk are still unknown. The hypothesized, but still unknown, mechanisms through which increased alcohol intake may favor the hormone-related growth of myomas are: (1) an increase of endogenous levels of estrogen through a reduction of estrogen metabolism [55]; (2) an interaction with a luteinizing hormone, with a consequent modulation of ovarian release of estradiol [56].

The BWHS also estimated the association between caffeine intake and uterine myomas. It did not report a relationship between caffeine consumption and uterine myoma risk overall, as confirmed by an Italian cross-sectional study [23]. Another investigation showed an increased risk among women aged <35 years [53]. A study examined the effect of caffeine consumption on ovarian hormones, showing that total caffeine consumption is associated with increased levels of early follicular-phase estradiol [57]. Caffeine inhibits phosphodiesterase and leads to the decreased clearance of cyclic adenosine monophosphates and through that mechanism enhances steroid production including female sex hormones [58]. It is also known that caffeine in high doses can induce stress-like effects in the hypothalamus-pituitary-adrenal axis, leading to the increased secretion of prolactin and a raised risk of uterine myoma [59,60].

There is a lack of studies regarding the possible association between tea consumption and uterine myoma risk, even if it is known that green tea extract leads to decrease of mouse uterine myoma cell proliferation as well as the decreased proliferation of human uterine myoma cell lines [61,62]. In fact, it was demonstrated that green tea extract and its active component, the epigallocatechin gallate (EGCG), in vitro inhibits proliferation and induce apoptosis in Eker rat leiomyoma tumor 3 cells and likewise in vivo exerts an inhibitory effect on human myoma cell lines [61,62], modulating catechol-O-methyltransferase (COMT) expression and enzyme activity [63]. The main epidemiological evidences belong to a double-blinded, placebo-controlled, randomized study on the efficacy and safety of green tea extract on uterine myoma and women’s quality of life. It was reported that uterine myoma volume increased in the placebo group, while significantly shrinking in the group who drank the green tea extract. It has also been reported that there was a significant reduction of uterine myoma-specific symptom severity and a significant improvement in the health-related quality of life in the group who drank the green tea extract. Thus, green tea extract could be an effective, safe, and inexpensive therapeutic agent in women with symptomatic uterine myoma [64].

### 3.6. Dairy Products and Uterine Myoma

There are conflicting results concerning the impact of milk and dairy products on the risk of myoma onset and growth. The results are controversial, with an Italian case-control study [23] indicating an absence of an association, but a Chinese prospective cohort study [27] indicating the opposite result. Their multivariate analysis verified that frequent milk consumption is an independent risk factor for uterine myoma [27]. Additionally, a study on African-American women reported a protective role of frequent milk and milk product intakes per day on myoma onset and growth, showing also a lack of association for ice cream, butter, and cheese consumption and a weak inverse association for yogurt intake [25]. In fact milk components, such as calcium and butyric acid, seem to have antiproliferative effects on myoma cells [65,66].

### 3.7. Vitamins and Uterine Myoma Risk

Vitamins are bioactive compounds, present in food in differing amounts, which have many physiological functions, however, to date their relationship with myoma risk and prevalence has not been adequately investigated. According to the Terry et al. [67] investigation, there is no link between carotenoids intake and the diagnosis of uterine myoma, but on the other hand, carotenoids increase the risk of diagnosing uterine myoma among current smokers. The BWHS showed an inverse association between dietary vitamin A and uterine myoma risk, but this was just for preformed vitamin A of animal origins and not for provitamin A from fruit and vegetable sources [22]. A cross-sectional study analyzed a total of 887 women aged 20 to 49 years, reporting a statistically significant dose-response relationship between vitamin A and uterine myoma occurrence, after adjustment were made for race, body mass index, education, and age [68]. The results obtained from animal model investigations concluded that uterine myoma formation occurs partly due to decreased vitamin A exposure, as vitamin A has an antiproliferative effect on uterine myoma [69]. Nowadays, as claimed by the literature, vitamin C, folate, and vitamin E do not influence uterine myoma risk [22,68]. Many studies focus on vitamin D serum concentration in relation to myomas. Baird et al. [70] reported that among American women aged 35 to 49 the group with normal vitamin D levels had an estimated 32% lower chance of myoma occurrence in comparison to the vitamin D deficient group, without distinction between African-American and Caucasian women [70]. In line with these results, Sabry et al. [71] found a statistically significant inverse correlation between vitamin D serum concentration and myoma, not only in terms of occurrence but also of volume. This latter association, once stratified for ethnicity, was confirmed only in African-American women but not in Caucasian women [71]. An analysis correcting outcome misclassifications by the National Health and Nutrition Examination Survey (NHANES) proved that the inverse association between myoma and vitamin D levels exists only in white women [72]. Both an Italian study and similarly an Indian study documented that women affected by myoma had a significantly lower vitamin D3 concentration in comparison to unaffected women, as well as a higher proportion of severe vitamin D3 deficiency in comparison to healthy women [73,74]. The mean concentration of vitamin D in the serum of women with uterine myoma was lower compared to controls. Moreover, there was a higher proportion of severely vitamin D-deficient women in a group of women with uterine myoma, in comparison with a group of women without uterine myoma [73,74]. Comparable results were obtained from further studies conducted in Indian, Turkish, and Chinese women, confirming again the correlation of vitamin D serum levels with uterine myoma risk [75,76,77]. An Italian clinical trial, in addition to confirming the correlation between the largest myoma for each patient and vitamin D serum levels, proved that vitamin D hypovitaminosis correction through supplementation in women with myomas reduced the need for surgical or medical treatment [78]. Literature data indicate that vitamin D has multiple roles in the modulation of uterine myoma growth. Vitamin D has an antifibrotic effect on uterine myoma via the reduction of transforming growth factor-beta3 (TGF*β*3) and extracellular matrix protein expression. It has also been reported that vitamin D inhibits uterine myoma cell growth through the downregulation of proliferating cell nuclear antigen and cyclin-dependent kinase 1 and the inhibition of COMT expression and activity. Additionally, it has been revealed that vitamin D regulates the expression and activity of matrix metalloproteinases, enzymes that play a role in extracellular matrix remodeling. Moreover, vitamin D presents potent antiestrogenic and antiprogesterone activities through reducing the expression of the estrogen and progesterone receptors. Other possible explanations as to how vitamin D decreases the growth of uterine myomas is its inhibition of wnt/β-catenin pathway activation and the role of vitamin D in DNA repair networks [79,80,81,82,83].

### 3.8. The Food Pollutants and Uterine Myoma Risk

Contamination of food by chemicals from the environment is an emergent and major global health, social, and food safety issue. The compounds which contaminate food belong to many various chemical groups. Some of these compounds occur naturally in the environment, while some chemical compounds originate from human sources. These chemicals exert adverse effects on our health. Thus, it will be necessary to perform estimations regarding the potential associations of these compounds and human disease in the future [84].

As explained above, myoma development is hormone-dependent and is mediated by the estrogen and progesterone receptors in the myometrium. Many contaminants harm women’s health by acting as endocrine-disrupting chemicals through their resemblance with endogenous steroid hormones, including estrogen and progesterone [85]. A Chinese case-control study revealed higher concentrations of phenolic environmental estrogens (bisphenol, nonylphenol, and octylphenol) in the urine and blood of the myoma group in comparison to the healthy group [86]. Conversely, the two Korean studies failed to prove that there is an association between bisphenol exposure and uterine myoma [87,88,89]. A higher urine concentration of phthalate metabolites (PMs) in myoma-affected women was demonstrated by the NHANES in the American population, in the Fibroids Observational Research on Genes and the Environment (FORGE) study, and in a Chinese study [89,90,91]. This latter, after adjusting for age, waist circumference, and parity, associated PM urinary concentration with higher uterine myoma risk [90]; additionally the FORGE study evidenced that PMs were positively correlated with fibroid size [91]. According to recent data of the FORGE study, PMs may influence myoma growth by miRNA epigenetic modification, as demonstrated by the eight phthalate-miRNA associations found in relation to race/ethnicity. In fact, mRNA gene targets of phthalate-associated miRNAs were significantly associated with angiogenesis, apoptosis, and proliferation of connective tissues, thus PMs ultimately favor myoma development [92].

Several studies investigated the association between early-life exposure to diethylstilbestrol (DES) and the onset of uterine myoma. It is known that early-life exposure to DES during a sensitive window of development can reprogram normal physiological responses and alter disease susceptibility later in life [93]. Baird et al. [94] reported a higher prevalence of uterine myoma in women who had been prenatally exposed to DES. This was confirmed by the Nurses’ Health Study II (NHSII) [95], conducted from 1989 to 2000 among women aged between 25 and 42 years old. The NHSII study evaluated the association between prenatal DES exposure and uterine myoma occurrence, evaluating that the first trimester of pregnancy, corresponding to the early stages of fetal Mullerian development, was the gestational period of DES exposure most at risk of myoma onset [78]. On a total of 1,273,342 person-years of follow-up, there were 11,831 cases of uterine myoma presence and the study concluded that women with prenatal exposure to DES have had a higher incidence of uterine myomas when compared with unexposed women [95]. The Sister Study has evaluated the associations between several early-life factors and the early onset of uterine myoma (early-onset uterine myomas were assessed based on the self-reporting of physician diagnosis of uterine myoma by the age of 30 years) in non-Hispanic white women. This study revealed that one of the factors most strongly associated with early uterine myoma was in utero exposure to DES [31]. Conversely, Wise et al. [96] refuted this thesis, only acknowledging a link between DES exposure and para-ovarian cyst formation.

### 3.9. Metaloestrogens and Uterine Myoma

There are not just organic compounds that influence uterine myoma risk. It has been revealed that heavy metals are associated with uterine myoma formation risk. A strong association with myoma risk has been reported for heavy metals, which are mostly present in smoking, polluted air, seafood, and leafy green vegetables. The Endometriosis: Natural History, Diagnosis, and Outcomes (ENDO) study showed a direct correlation between uterine myoma diagnoses and increased serum levels of cadmium and lead and urinary cobalt levels, also proving the contribution of these trace elements to myoma growth [97]. The evidence of Korean women assessed showed no association between myoma and blood concentrations of heavy metals, however high serum concentrations increased the chances of having myoma. Cadmium serum levels were significantly associated with myoma size [98]. Heavy metals may increase uterine myoma risk as they activate both the estrogen receptor in the absence of estradiol as a metaloestrogen [99,100] and influence the hypothalamic-pituitary-ovarian axis as endocrine-disrupting compounds [101]. Myoma-affected women presented higher levels of serum copper and chrome and lower serum levels of zinc in a Chinese study, and lower levels of serum selenium in a Bulgarian study in comparison to the healthy control groups [102]. In the Japanese quail, commonly used as animal model of uterine myoma, supplementation with selenium and zinc reduced the incidences of spontaneously occurring myomas of the oviduct, thus suggesting a possible human benefit from selenium and zinc supplementation [103,104].

## 4. Conclusions

Our review of the literature led to these conclusions: a low intake of fruit and green vegetables is linked to a higher risk of myoma formation; vitamin D deficiency is associated with an increased risk for uterine myoma onset; pollutants ingested with food increase uterine myoma risk. It is unclear how exactly specific dietary products and nutrients influence uterine myoma onset. Future studies that control numerous potential confounders and consider different food content, environmental exposures, and ethnicities are necessary for further understanding of myoma biology and pathophysiology.

## Figures and Tables

**Table 1:** Food types and their influence on uterine myoma risk.

Food	Related Investigations	Influence on Uterine Myoma
Whole food	He, Y. et al. [20], Nagata, C. et al. [21], Wise, L.A. et al. [22], Chiaffarino, F. et al. [23]	No significant association
Soya	He, Y. et al. [20], Nagata, C. et al. [21], Atkinson, C. et al. [24], Wise, L.A. et al. [22]	No significant association
	Shen, Y. et al. [26], Gao, M. et al. [27]	Increased the risk
Dietary fat	He, Y. et al. [20], Nagata, C. et al. [21], Chiaffarino, F. et al. [23]	Increased the risk
Meat	Chiaffarino, F. et al. [23]	Increased the risk
	He, Y. et al. [20]	No significant association
Fish	Lambertino, A. et al. [40]	Increased the risk
Fruit	He, Y. et al. [20], Wise, L.A. et al. [22], Shen, Y. et al. [42]	Decreased the risk
Vegetables	He, Y. et al. [20], Shen, Y. et al. [42]	Decreased the risk
Alcohol	Wise, L.A. et al. [53], Templeman, C. et al. [54]	Increased the risk
	He, Y. et al. [20], Chiaffarino F et al. [23]	No significant influence
Coffee	Wise, L.A. et al. [53]	Increased the risk
	Chiaffarino, F. et al. [23]	No significant influence
Dairy products	Wise, L.A. et al. [25]	Decreased the risk
	Gao, M. et al. [27]	Increased the risk

**Table 2:** Natural compounds and their influence and possible mechanism of action on uterine myoma.

Natural Compounds	Influence on Uterine Myoma	Mechanism of Action
Strawberry extract Islam, M. et al. [44], Giampieri, F. et al. [45]	Increase apoptosis of uterine myoma cells	Increases oxygen species. Decreases extracellular acidification rate, activin collagen 1A1, fibronectin, versican mRNA expression
Curcumin Malik, M. et al. [46], Tsuiji, K. et al. [47]	Increase apoptosis of uterine myoma risk	Decreases fibronectin production, stimulating caspase-3 and caspase-9 expression; inhibits extracellular signal-regulated kinase 1 (ERK1) and ERK2 and nuclear factor kappa B (NF-κB); activates of peroxisome proliferator-activated receptor-γ
Quercetin and indole-3-carbinol Greco, S. et al. [48]	Antiproliferative effect	Reduces expression of fibronectin collagen 1A1
Lycopene Sahin, K. et al. [49], Sahin, K. et al. [50]	Decreases size and incidence of uterine myoma	Modulates the expression of cell cycle regulatory proteins, modulates the IGF-1/IGFBP-3 system, up-regulates tumor suppressor protein Cx43, increases gap junctional intercellular communication, modulates re-dox signaling, prevents oxidative DNA damage, modulates carcinogen-metabolizing enzymes.
Resveratrol Ho, Y. et al. [51], Chen, H.Y. et al. [52]	Decreases myoma cell proliferation	Modulates cross-talk between integrin αvβ3 and IGF-1R, increases expression of cyclooxygenase (COX)-2, p21 and CDKN2, decreases expression of fibronectin, proliferates cell nuclear antigen α-smooth muscle actin, upregulates the ratio of Bcl-2-associated X and B-cell lymphoma/leukemia 2 in vivo collagen type 1 and α-SMA and protein levels of β-catenin
